# Multidisciplinary Approach to Agricultural Biomass Ash Usage for Earthworks in Road Construction

**DOI:** 10.3390/ma15134529

**Published:** 2022-06-27

**Authors:** Ivana Barišić, Ivanka Netinger Grubeša, Davorka K. Hackenberger, Goran Palijan, Stella Glavić, Marija Trkmić

**Affiliations:** 1 Faculty of Civil Engineering and Architecture Osijek, Josip Juraj Strossmayer University of Osijek, Vladimira Preloga 3, 31000 Osijek, Croatia; 2 Department of Biology, Josip Juraj Strossmayer University of Osijek, Cara Hadrijana Street 8/A, 31000 Osijek, Croatia; davorka@biologija.unios.hr (D.K.H.); gpalijan@biologija.unios.hr (G.P.); stella.glavic@biologija.unios.hr (S.G.); 3 Central Laboratory for Chemical Technology, HEP Group, Zagorska 1, 10000 Zagreb, Croatia; marija.trkmic@hep.hr

**Keywords:** biomass, biomass ash, earthworks, road construction, environment, heavy metals

## Abstract

Agricultural biomass has great bioenergy potential due to its availability, and it is a carbon-free energy source. During biomass incineration, biomass ash is formed, which is still considered as a waste without proper disposal and management solutions. Various biomass ash utilization options were investigated, mainly concerning engineering issues (the mechanical characterization of newly produced building materials or products), and there is a lack of knowledge of environmental issues arising from this “waste” material utilization in civil engineering practice. The main aim of this research is discussion of a different agricultural biomass characteristics as a fuel, the impact of agricultural biomass ashes (ABA) on the mechanical properties of stabilized soil with a particular emphasis on the environmental impacts within this kind of waste management. The results of this study indicate improved geotechnical characteristics of low-plasticity clay stabilized by lime/ABA binder. In addition to mechanical characterization for materials embedded in road embankments and subgrades, appropriate environmental risk assessment needs to be performed, and the results of this study indicate that the amount of ABAs added to the soil for roadworks should not have adverse effects on the soil fauna in the surrounding environment.

## 1. Introduction

We live in a fossil fuel era with 80% of global energy and around 64% of global electricity still being produced by fossil fuel, including coal, oil, and natural gas [1,2]. Electricity generation from fossil fuels increased from year 1985 to 2020 by 151% while annual CO_2_ emissions increased in 372% during the same period [2]. Some globally major sources of CO_2_ emissions are power plants operating on fossil fuels, and there is an urgent need to shift energy production technology to sustainable and renewable energy sources. During the last 10 years, renewable energy technologies had a share of 13–14% in total primary energy supply globally, while biomass provides around 10% of all renewable electricity generation [3].

The applicability of biomass as an energy resource lies in its availability and the fact that it presents a carbon-free energy source. Particularly, the agriculture sector has a huge bioenergy potential and additional value due to both fuel and food production. However, biomass ash as a biomass combustion product is still considered a waste, and its proper disposal and management is a concern.

Agricultural biomass is a residue in the fields after harvesting crops, which is then used to produce energy in biomass power plants, during which, ash is generated. However, not all produced biomass can be utilized for energy purposes. In e view of sustainable agriculture development, biomass removal rates are 40–50% depending on the cultivated crop [4], and the rest should be left on agricultural soil for ploughing, erosion prevention and for the increase in soil carbon content [5]. The most appropriate and sustainable method of biomass disposal would be to use it as an energy source, as suggested earlier. The amount of agricultural biomass ash (ABA) arisen from agricultural biomass combustion depends on the content of organic and inorganic matter and possible impurities, sampling point, harvesting time and harvest conditions [6].

Utilization of biomass ash as soil amendment and fertilizer (due to present abundant potassium and useful phosphate [7]) is indicated to be the most ecological and sustainable disposal method due to mechanism of returning the macro- and micronutrients taken by the plants back to the soil [6]. However, new possibilities are explored and one of such, ABA utilization in road construction is also presented within this research.

Civil engineering essentially relies on using natural materials, and road construction is an activity that uses large amounts of electrical power and energy from non-renewable sources. Road construction and exploitation also result in large amounts of waste, water contamination and air pollution. This especially applies to materials and the method of constructing and maintaining the road bed, which is in direct contact with the environment, where potential contamination can easily spread. Therefore, it is necessary to reduce the negative impact of road construction on the environment through modernizing the production processes and the origin of basic materials, the road construction process itself and the methods of maintaining already built road networks.

The advantage and potential of the ABA application in road construction lies in the large quantities of materials that are incorporated in roads compared to other branches of civil engineering. In addition, due to the chemical reactions of clay and ash minerals, there is a potential to produce a better quality pavement layer than with the usual lime stabilization. Variable ash composition, which depends on a variety of factors, is identified as a fundamental potential drawback that makes it difficult to standardize and prescribe an exact composition of the soil-lime-ABA mixture for practical application.

Biomass ash utilization in road works has numerous studies [8,9,10,11,12] dealing with the different biomass ash types used in different pavements and road parts. Depending on its characteristics, ash can be utilised as a filler, fine-aggregate fraction replacement, binder supplement or as a binder itself [13]. In order to behave properly during the entire design period, good quality road subgrade is of high importance. Locally available soil is often not suitable for subgrade or embankment construction due to its low bearing capacity; therefore, biomass ash has been researched for soil improvement and as a stabilizing agent.

Biomass ash potential application is primarily dependent on its chemical composition. Pulp mill fly ash is rich in calcium, and thus it can be used as stand-alone binder for long-term soil stabilization [14]. Soil expansion reduction can be achieved by the use of bottom ash from biomass (olive) combustion to the same extent as from lime treatment [15]. Using mango kernel ash as fruit processing industrial waste with calcium carbide residue and lime significantly changed the expansive soil properties by decreasing its plasticity and improving the CBR (California Bearing Ratio) and UCS (Unconfined Compressive Strength) values [16].

Combinations of rice husk ash and calcium carbide residue from acetylene plants were used for the stabilization of expansive soil with excellent performance, lowered construction and disposal costs and reduced environmental pollution [17]. Soil improvement in a view of CBR and UCS improvement and the control of soil volumetric change is possible using rice husk ash and sugarcane bagasse ash [18].

Similar results were presented in [19] where a mixture of rice husk ash and sugarcane bagasse ash combined with lime as an activator resulted in an increased UCS. The combination of wheat husk and sugarcane straw ash also has a positive effect on soil geotechnical properties [20]. When analysing the application potential of any waste or alternative material within road construction, particularly for earth works, it is essential to evaluate all potential environmental risks [21,22]. Biomass ash application within road construction with a view of environmental risk is mainly discussed regarding the heavy metal content and leaching potential.

Sources of heavy metals in agricultural biomass are fertilizers and minerals presented in soil and water, while a significant rate (70–90% [23]) of biomass presents heavy metals that pass to biomass ash during the combustion process. Although was found that ABA contains high amounts of K, modest amounts of phosphate, and very low levels of heavy metals [7], the contents of Cd and Pb in certain fly ash samples exceed the limit values for agricultural soil and have a high leaching rate of K [24]. The leaching rate is dependent on the environment (soil) pH [25] where a lower soil pH lead to increased heavy metal activity [26] and moderate or low solubility at a neutral and moderate acidic pH [27]. Decreased leachability can be achieved by a hydration process or cementation process in soil stabilized by biomass ash [14] or in cement paste and mortars up to 30% [28].

Leached material could have different effects on soil organisms. The activity of soil bacteria could be decreased but also increased by ABA, while effects are concentration-dependent. Similarly, the application of different biomass ashes affected earthworm and collembolan survival and reproduction depending on the soil type and the amount of ashes [29]. Apart from the risk of heavy-metal accumulation, soil organisms are sensitive to an increased pH caused by a high content of oxides and hydroxides in ashes [30]. Hence, the effects of a particular ABA should be investigated before its application in the environment.

In [31], within the analyses of potential biomass ash application for geotechnical purposes, the need for increasing cooperation between scientific research institutions and biomass power plants promoting the interdisciplinary integration of machinery, chemistry, materials, rocks and soil and the environment was presented. Thus, the purpose of our presented research is to promote potential ABA application in road construction aiming to provide additional value to the topic through a multidisciplinary approach.

The experimental study of the properties of seven biomass ashes used as additives to lime-stabilised low-bearing soil for embankment and subgrade purposes is presented. The scope of this research is the discussion of different agricultural biomass characteristics as fuel, the impact of ABAs on the mechanical properties of stabilized soil and a special emphasis on the environmental impacts in order to promote sustainable road construction development and a circular economy.

## 2. Materials and Methods

### 2.1. Biomass and Biomass Ash

This research analysed four agricultural types of biomass (soy straw—SS, sunflower seed husks—SH, wheat straw—WS and barley straw—BS) for potential fuel applications and combustion residues (ABA) for potential road work applications. In Table 1, the biomass characterization is presented. The biomass samples were first dried, and their total moisture was determined. Later, samples were ground in a mill to less than a 1 mm particle size. The ash, sulphur and chlorine contents and calorific values were determined. Determination of the ash content was performed gravimetrically in a muffle furnace, and based on this result, it is possible to estimate the amount of ash that will be produced by burning biomass in power plants. Although sulphur and chlorine contents in biomass are found in very low concentrations, during fuel combustion, they can cause corrosion on the exchange surfaces of the furnace; therefore, these are important data in estimating fuel potential.

The calorific value is the amount of thermal energy obtained by burning biomass in a power plant, and this was determined experimentally by a calorimeter. The calorific value is mainly affected by the chemical composition and moisture of the biomass. The gross calorific value (GCV) is the amount of heat generated by the complete combustion of a unit amount of fuel, where the flue gases are cooled to 25 °C, and the moisture from the flue gases is removed as condensate. The net calorific value (NCV) is the amount of heat generated by the complete combustion of a unit amount of fuel, where the flue gases are cooled to 25 °C, the moisture in the flue gases remains in a vapour state, and the heat of condensation remains unused.

**Figure 1:**
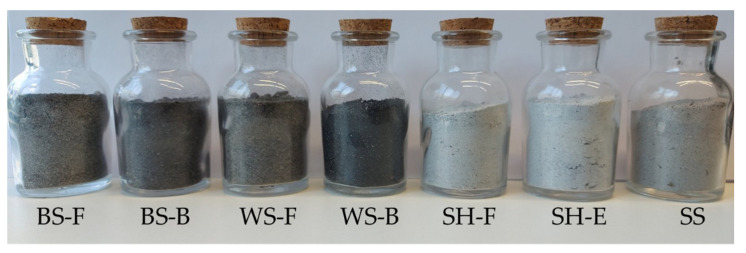
Different types of ABA.

Within this research, seven ABAs were analysed (Figure 1) arising from four different types of biomass. Sunflower seed husk ash was taken from the industry (sunflower oil factory), where two types of ashes are formed: fly ash (SH-F) and economizer (heat ex-changer) ash (SH-E). Within the same factory, trial attempts were made for using BS and WS as fuel, and two ashes of each biomass were formed: fly ash (F) and bottom ash (B). Soy straw ash (SS) was taken from a household that uses soy straw as a heating energy source. All four types of biomass were also taken to the laboratory, and laboratory ash samples were prepared by combustion in a muffle furnace for further chemical analyses. All ash samples were sieved to a particle size of less than 50 μm and pelletized on a hydraulic press. The ash pellets were analysed by an EDXRF spectrometer. The chemical composition of the ashes determined according to ISO/TS 16996 [30] are shown in Table 2 and Table 3.

The analyses of agricultural biomass as a fuel must include potential risk assessment for the used equipment (boiler, power generation equipment and furnace), which arise from potential slagging, corrosion and ash deposition [32]. The slagging tendency of biomass fuel during combustion can reduce the combustion efficiency, and it is predicted by empirical indices or the empirical index discrimination calculated on the basis of biomass ash chemical composition. In [32], detailed analyses of various slagging empirical indices were analysed indicating that the specific slagging situation cannot be accurately predicted only based on a single index. Thus, three additional indexes are used together with the alkali index (Al), which is known as a reliable slagging identification method [32,33], and the results are presented in Table 4.

### 2.2. Stabilized Soil

**Figure 2:**
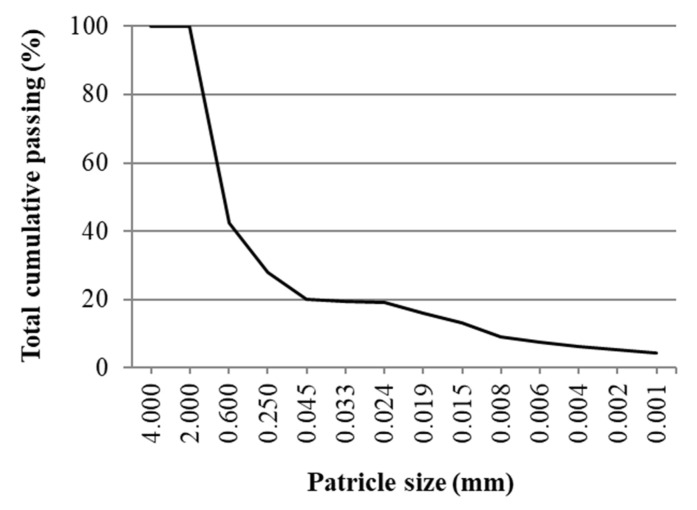
Soil particle size distribution curve.

The potential of ABA application in road construction was analysed for partial lime substitutes within lime-stabilised weak soil for road embankment and subgrade construction. Low-plasticity clay CL according to USCS (Unified Soil Classification System) classification was used as the base material with liquid and plastic limits of 34.5% and 21.9%, respectively and plasticity index of 12.5% determined according to standard EN ISO 17892-12 [34]. The size distribution of used soil (Figure 2) was determined according to standard EN ISO 17892-4 [35] by combination of sieving and hydrometer methods. The optimal water content and maximal dry density, determined by standard EN 13286-2 [36], were 13% and 1.80 g/cm^3^, respectively. As a stabilising agent, CL 80 S hydrated calcium lime was used according to EN 459-1 [37] with a density of 2.65 kg/dm^3^.

The density of used materials was determined by EN 1097-7 [38], and the specific surface area (SSA) was determined using the BET method according to standard ISO 9277 [39] (Table 5).

The optimal lime and ash portions were determined by standard ASTM D 6276-99a [40], measuring the pH values of soil–lime and soil–lime–ash mixtures with various content ratios. The optimal lime content was 7% of the total dry mass of soil, and the optimal lime/fly ash ratio was 80/20%, while the optimal lime/other ash ratio was 70/30%. For lime- and lime/ABA-stabilised soil, the maximum dry density (MDD) and optimal moisture content (OMC) were determined according to standard EN 13286-2 [36] with standard Proctor compaction energy. The specimens were prepared at their respective OMC and MDD, measured after compaction to 100 mm in diameter and 120 mm in height.

The prepared specimens were cured for 28 days in a temperature and moisture-controlled chamber (20 °C and 60% relative humidity). A compressive strength test (according to standard EN 13286-41 [41]) was analysed. More information and results of here used ABAs for stabilised soil purpose can be found in [42]. Previous research has proven possible ABAs usage in road construction considering mechanical properties of lime–ABA-stabilized soil. The main purpose of our presented research is a multidisciplinary approach to feasibility assessment of agricultural biomass as a fuel and ABAs environmental influence when inbuilt in a road embankment or subgrade.

### 2.3. Heavy Metal Content

The total heavy metal concentration in ABAs taken from industry was determined according to ISO standards: arsenic according to EN 14332 [43] and cadmium, chromium and lead according to EN 14084 [44]. In addition to ABA classification as a waste material based on total contaminant concentrations measured in the solids, a leaching test was also conducted according to EN 12457-2 [45] and EN 15586 [46] since the leaching properties correspond directly to the risk of potential release of harmful substances from the material to groundwater or soil.

### 2.4. Risk Assessment of ABA Leachate on Soil Organisms

The same eluates from ABAs were prepared for experiments on both earthworms and microorganisms. They were prepared with the optimal lime and ash ratios obtained within this research (lime: fly ash 80:20 and lime: economizer/furnace heart ash 70:30). For the reference only lime was added, and as a negative control only distilled water was used. Namely, natural soil was mixed with appropriate amount of lime and ABA and water. This mixture was set on a rotary shaker for 24 h and then filtered. The eluates were stored at 4 °C until usage in the experiments. The pH values and conductivity of eluates were measured in the preliminary experiment, while for the final experiment pH (in KCl) and conductivity were measured in a moistened artificial soil.

#### 2.4.1. Mortality and Molecular Biomarkers in Earthworm (*Eisenia fetida*)

Earthworms (*Eisenia fetida*) were obtained from a culture maintained at the Department of Biology, University of Osijek. All earthworms weighted between 500 ± 300 mg. Prior to each exposure, earthworms were removed from their culture and left on moist filter paper in dark environment to void the gut content.

In the preliminary experiment the earthworms were exposed on a filter paper to ABA eluates according to an OECD 207 protocol [47]. The period of exposure was 48 h, after which survival was assessed. According to the results of a preliminary experiment, ABAs that had a mortality rate higher than a reference eluate (only lime) were selected for a final experiment. Namely, those were WS-F, WS-B, BS-E, BS-F and SH-E. In the final experiment, the earthworms were exposed to a four concentrations of selected ABAs eluates (100%, 75%, 50% and 25%) obtained by mixing with distilled water to gain a 50% water holding capacity of standardised artificial soil [48]. The earthworms were weighed, and a batch of five individuals were added to a glass vessel with an artificial soil moistened with different concentrations of ABA eluates.

After seven days of exposure, the earthworms were removed, washed in distilled water and left to void their gut content for 24 h. After that the earthworms were individually weighted and homogenised in a cold sodium phosphate buffer (0.1 M, pH 7.2), in 1:5 w:v ratio with a Potter–Elvehjem homogeniser. The homogenates were centrifuged at 9000× *g* at 4 °C for 30 min to yield the postmitochondrial fraction (S9). The obtained supernatants (S9) were stored at −80 °C until further analysis. Several molecular biomarkers were measured in order to assess the effects of ABA eluates on earthworms: acetylcholinesterase (AchE) activity [49], glutathione S-transferase (GST) activity [50], superoxide dismutase (SOD) activity [51] and lipid peroxidation determined by measuring the formation of thiobarbituric acid reactive substances (TBARS) [52]. The total content of proteins per organism was detected using the Lowry method [53].

#### 2.4.2. Activity of Soil Microorganisms

To investigate the effects of ashes on microbial enzyme activity and biofilm-forming ability natural soil was exposed to ash leachate. For that purpose, microcosms were assembled consisting of sterile glass test tubes (16 mm × 100 mm, with a metal cap) and 3 g +/− 0.05 g of the soil sample. To ensure thorough wetting of the soil in the microcosm, 1 mL of leachate was added to the top of the soil resulting in 85% saturation of the water holding capacity.

All treatments were replicated five times and were incubated for 7 days at 25 °C. The experiment consisted of seven treatments, a reference and a control. The control microcosm was wetted with sterile distilled water, while the reference microcosm was wetted with leachate from the mixture of the soil and lime. Treatments consisted of a mixture of soil with lime and ash. After incubation, from every microcosm 600 mg +/− 10 mg of soil was weighed and placed in a 2 mL sterile plastic centrifuge tube and mixed with 1 mL of sterile distilled water. Mixtures were vortexed for 10 s at 1000 rpm to homogenize the soil.

Biofilm forming ability (BFA) was determined with a crystal-violet test with the whole soil suspension as inoculate [54] in clear, polystyrene, flat-bottom 48-well plates. In each well 100 µL of M63 minimal media supplemented with 2 mg/L glucose, 1:1000 dilution of the nutrient broth (final concentrations) was added. This was followed by 100 µL of soil suspension added with wide orifice pipette tips. The first and last well plate columns were filled with 200 µL of sterile distilled water.

The plates were incubated for 24 h at 25 °C. After incubation, the well plates were washed with tap water to remove soil and unbound material by submerging the whole plate in a plastic container filled with water. After washing, the plates were stained with crystal violet solution (0.1%) for 15 min at room temperature. The excessive stain was washed three times, and after air-drying, the stain was dissolved in 1 mL of 96% ethanol per well and measured at 588 nm with a microplate reader (Spectrostar Nano, BMG Labtech, Ortenberg, Germany).

The effects of the ash leachate on the hydrolytic microbial activity were assessed by the fluorescein diacetate test [55]. From each soil sample, 200 μL of suspension was transferred in a new 2 mL sterile centrifuge tube and supplemented with 800 μL of 75 mM, pH = 7.6 phosphate buffer and 40 μL of 250 μL/mL FDA solution. The samples were vortexed, protected from light by aluminium foil and incubated on a rotary shaker for 30 min at 30 °C. The incubation was terminated by adding 800 μL of chloroform: methanol mixture, centrifuged at 10,000 rcf and 20 °C for 5 min, and immediately measured at 485 nm with UV-Vis spectrophotometer (Spectrostar Nano, BMG Labtech).

Leachate effects on the dehydrogenase activity (DHA) were determined according to ISO standard [56] with few modifications. The remaining soil in 2 mL centrifuge tube was supplemented with 450 μL of TRIS buffer (100 mM, pH 7.6) and 225 μL of 2-(4-iodophenyl)-3-(4-nitrophenyl)-5-(phenyl) tetrazolium chloride (INT) 0.12% solution. After vortexing for 10 s, the tubes were covered with aluminium foil and left for incubation for 24 h-period at 30 °C. After incubation, the tubes were centrifuged, supernatants were discarded and replaced with 1 mL of acetone. The newly made mixture was vortexed. After one hour of extraction, samples were vortexed and centrifuged again. The obtained supernatants were measured at 485 nm with a UV-Vis spectrophotometer (Spectrostar Nano, BMG Labtech).

## 3. Results and Discussion

### 3.1. Characterstics of Agricultural Biomass and Agricultural Biomass Ash

As presented in Table 1, barley straw (BS) and wheat straw (WS) created the highest ash content (particularly in as received state), which is not a favourable fuel property. The measured calorific values present good potential of using agricultural biomass as a fuel with net calorific value ranging from 17.25 MJ/kg to 20.01 MJ/kg, which are comparable to that of coals (~27 MJ/kg) or wood chips (~18 MJ/kg). Sunflower seed husk (SH) is suggested as a favourable fuel source due to the low ash content and high calorific values.

Ash content is dependent on biomass itself (organic and inorganic matter and possible impurities) and on the sampling point, harvesting time and conditions [6]. Slagging tendency for SH is also highest (Table 5) and caution is needed when considering its introduction as a fuel. Slagging tendency predicted by empirical indices is showing highest slagging and fouling potential for SH while lowest for BS. The results of a different empirical indexes are showing similar trends among tested biomasses with only Al (alkali index) presenting high risk for all tested biomasses.

**Figure 3:**
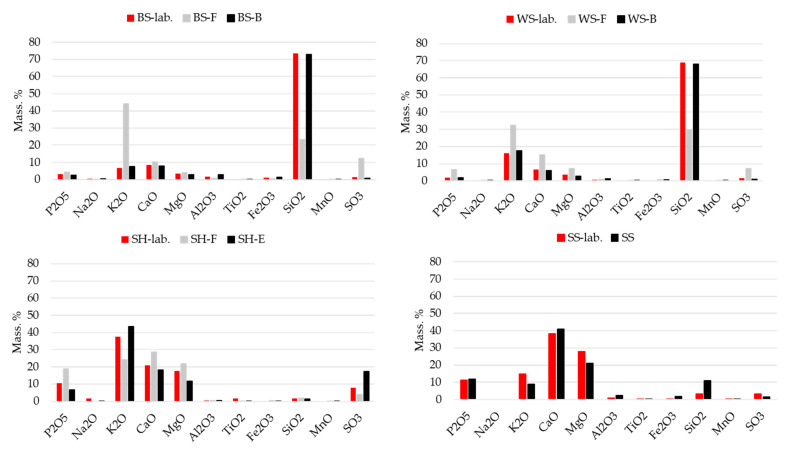
ABA chemical composition.

Monitoring the ash composition plays an important role in considering biomass combustion and its potential use as a fuel. From the results presented in Table 2 and Table 3 and Figure 3, it is evident that there is a difference in ash chemical composition depending on agricultural biomass type but also on ash origin (industry or laboratory biomass combustion). Within ABA’s chemical composition, potassium (K_2_O), calcium (CaO), silicon (SiO_2_) and magnesium (MgO) oxides are dominant in different ratios. Calcium and magnesium oxides increase the melting points of ash, while potassium oxide decreases them because silicon with potassium forms low-melting silicates in fly ash.

Due to the relatively large amounts of potassium, ABA is sintered and melted at significantly lower temperatures compared to the ash of wood fuels. In combination with chlorine and sulphur, potassium and sodium play a significant role in corrosion mechanisms. These elements partially evaporate during combustion and form alkaline chlorides that condense on the exchange surfaces of the furnace and react with the flue gases to form sulphates and release chlorine. High potassium content influence higher slagging emerge, and contrary, less alkali and high calcium contents results in a more manageable slagging, fouling and corrosion occurrence within a combustion furnace [6]. It is in accordance with empirical indices presented in Table 4, where the highest potassium level recorded in SH ash (Figure 3) presents the highest slagging tendency according to all empirical indices.

The results presented in Figure 3 also show different chemical compositions for different ash origins (laboratory ash obtained from combustion in a muffle furnace and real ash samples obtained from power plants. As indicated in [6], the chemical composition of biomass ash depends on biomass itself and the process of combustion (fuel preparation, combustion technology, combustion conditions) and other technical conditions. However, laboratory-produced ashes have similar content as bottom ash, while fly ash has higher amounts of potassium, calcium and manganese oxides. Finally, possible ABA use for civil engineering purposes is dependent on its chemical composition, every ash needs to be analysed separately before defining its potential use, and it is unlikely that general recommendations will be defined.

### 3.2. ABAs Influence on Stabilized Soil

The addition of lime and ABAs to low-plasticity clay (CL) resulted in plasticity index decreases (from 12.48% of CL to the lowest 10.60% for WS-F), which presents soil improvement in terms of higher stability and less swelling affinity, which is in line with results presented in [18]. According to Proctor test, all lime/ABA mixtures show similar sensitive to moisture deviation, with OMC of 18% and MDD of 1.6 g/cm^3^. Lime replacement by ABA resulted in an increase in OMC and a decrease in the MDD comparing to simple lime-stabilised soil (OMC = 16% and MDD = 1.7 g/cm^3^) and pure soil (OMC = 13.30% and MMD = 1.80 g/cm^3^).

This also presents an improvement in the soil characteristics, since earth works may be performed with more moisture in soil during the rainy season (after rainy days). Increased moisture in the soil is in fact a major problem when carrying out earthworks, as it is not possible to achieve the necessary compaction. When mixing soil and lime, and as presented in this research ABA, chemical processes and changes in the soil structure occur, which manifest themselves in an increase in the optimal soil moisture, thus enabling construction works with a wider range of material moisture. The decrease in MDD may be due to the ABA with lower density and higher SSA is substituting higher density and lower SSA lime (Table 5) [18,20].

**Figure 4:**
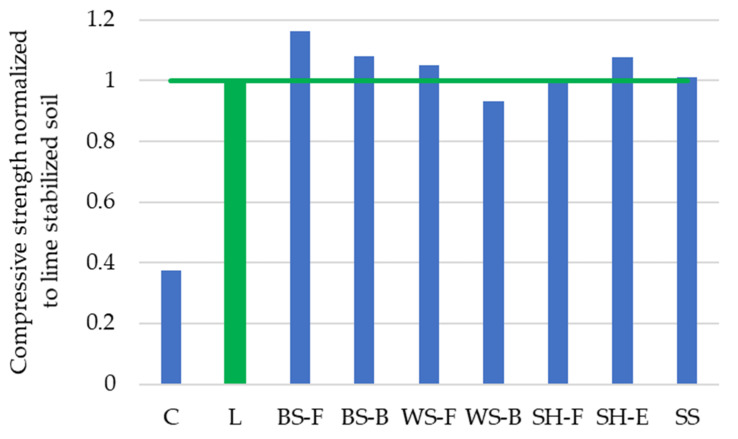
Normalized lime/ABA-stabilized soil compressive strength results.

From the normalized results of compressive strength (comparing to lime stabilized soil having compressive strength of 0.78 MPa) presented in Figure 4, it can be concluded that substituting 20–30% of lime by ABA has little influence on stabilized soil strength. Obtained strength are comparable to that of lime stabilized soil with BS-F having the highest, 16% higher compressive strength and WS-B having the lowest, 7% lower compressive strength. All tested mixtures had compressive strength above 0.5 MPa (0.73 MPa for WS-B to 0.91 MPa for BS-F), which is satisfactory for general road subgrade and embankment purposes.

Comparing the results from Figure 4 and ABA chemical composition presented in Figure 3, different mechanisms of strength development can be observed. Barley straw ashes are rich in K_2_O and SiO_2_, which contributes to the development of strength through an alkali silicate (K_2_O) and pozzolanic reaction (SiO_2_, particularly its reactive form [57]). Sunflower seed husk and soya straw ashes are rich in free or reactive CaO and MgO but low in SiO_2_ and hydration of these elements contribute to strength development without additional pozzolanic reaction.

More information, results and discussions of the used ABAs for stabilised soil purpose can be found in [42], while the main purpose of our presented research is the feasibility assessment of agricultural biomass as a fuel and the ABA environmental influence when inbuilt in road embankment or subgrade as additional value to the research of ABA use in road construction.

### 3.3. Heavy Metals and Leaching Potential

**Figure 5:**
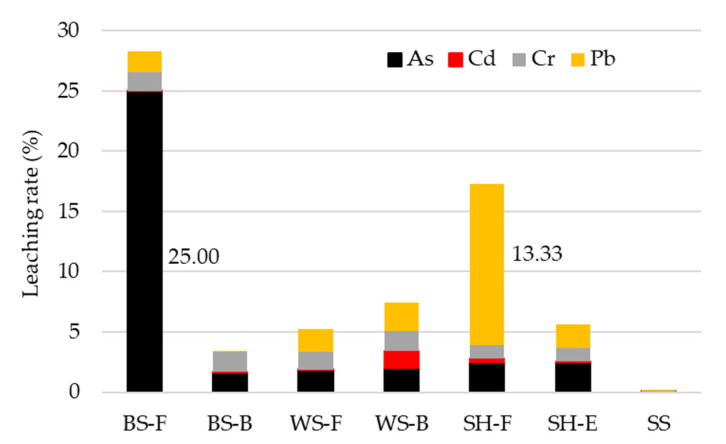
Leaching rates of heavy metals from ABAs.

The total heavy metal content and leaching rates are presented in Table 6 and Table 7 and in Figure 5.

Based on Croatian regulation [58], which is in accordance with European Directives dealing with wastewater treatment, the emission limits of heavy metal pollution in surface wastewater are shown in Table 8. EPA United States national regulatory standards for wastewater discharged to surface waters and municipal sewage treatment plants is presented for the daily maximum limits of the World Health Organization from a year 2006 (WHO).

As presented in Table 6, dominant heavy metal in researched ABAs is chromium (Cr) ranging from 8.41 mg/kg (BS-F) to 83.5 mg/kg (SH-F). The leaching rate of Cr is low, up to 1.7% but even at this low leaching level, high amounts of this element are released to the environment (0.94 mg/L for SH-F), and these exceed the limits presented in Table 8.

Cadmium (Cd) is one of the most toxic elements with adverse influence on living organisms [23], and its content is higher in fly ashes compared to bottom ash of the same biomass. However, the total leached Cd content is very low, ranging from 0.01% (WS-F) up to 1.45% (WS-B).

Arsenic (As) is also a highly poisonous and toxic heavy metal adversely influencing all living organisms and that can contaminate water sources [23,61]. Within the researched ABAs and heavy metals, lowest levels of arsenic in ABAs are recorded (0.04–1.26 mg/kg); however, it also has the highest leaching rate (0.05–25%). Generally, As is present in more rate within ash from furnace combustion parts (bottom and economizer) than in fly ash.

The presence of carcinogenic and mutagenic lead (Pb) is also analysed, and the highest rate is recorded in SS ash. However, the lowest leaching rate is also recorder for SS (0.03%) and second highest leaching rate of all analysed heavy metals is recorded for SH-F (13.33%). The total leached Pb is however low (0.003–0.01 mg/L) and does not exceed permitted limits from Table 8. Generally, ABAs have highest total amount (in mg/kg) and leached amount (in mg/L) of Cd; however, the most mobile element present in all ABAs are Pb and As with highest leaching rates (in %). The maximum amount of leached heavy metals can not be predicted (represented) by its total content in ABA since some elements can easily change into their stable, more difficult to leach states [28,62].

### 3.4. Mortality and Molecular Biomarkers in Earthworm (Eisenia fetida)

The preliminary experiment with a filter paper test does not represent an environmentally relevant exposure scenario but instead gives an insight in magnitude of effect between different substances. The BS-E ash caused the highest mortality (60%), followed by SH-E, BS-F, WS-F and WS-B with 40% (Table 9). Only BS-B did not cause any mortality, while SH-F and SS had the same effect on survival as the treatment with lime only (20% mortality).

Both conductivity values and pH were very high and in the toxic range for earthworms as *Eisenia fetida* can tolerate pH values between 4 and 9; however, already above 7, the inhibition of reproduction has been observed [63]. However, as in all treatments these values were high and mortality rate varied, some other factor was responsible. Additionally, as no correlation was found between a particular heavy metal content and mortality, the conclusion is that the mortality occurred due to the combined effect of osmotic stress and mixture of different heavy metals and oxides.

As the application of lime is a common process of soil stabilization for roadworks, we considered all the ABAs that had mortality equal to or lower than the treatment with lime adequate for environmental application and did not test them further. However, for ABAs that had a higher mortality, we had conducted a final experiment in a more environmentally realistic scenario.

**Figure 6:**
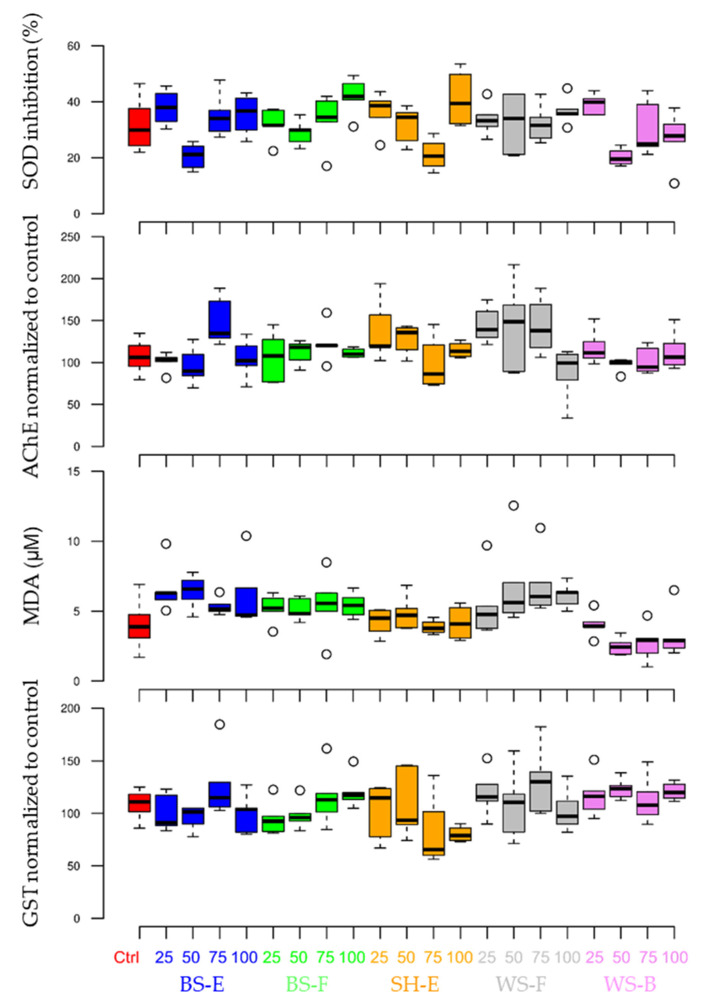
Measured biomarker values in earthworm *Eisenia fetida* after seven-day exposure to different ratios of ABAs in an artificial soil test.

After seven days of exposure, no mortality occurred in any treatment. Additionally, no significant differences in measured biomarkers between the control and ABA treatments were observed (Figure 6). The pH of the soil in treatments was in the optimum range, while the conductivity was only moderately higher and did not cause osmotic stress to the earthworms. The amount of ABAs that would be added for soil stabilization in roadworks and could be leached into the surrounding soil should not have an adverse effect on soil fauna. This results is in accordance with some previous studies were the effect of forest ash had an effect on survival and reproduction of earthworm *E. andrei* only at the dose of 20 tha^−1^ [29], or the *Miscanthus* ash that had a lethal effect on earthworm *Aporrectodea caliginosa* at application rates up to 10 tha^−1^ [64].

### 3.5. Activity of Soil Microorganisms

**Figure 7:**
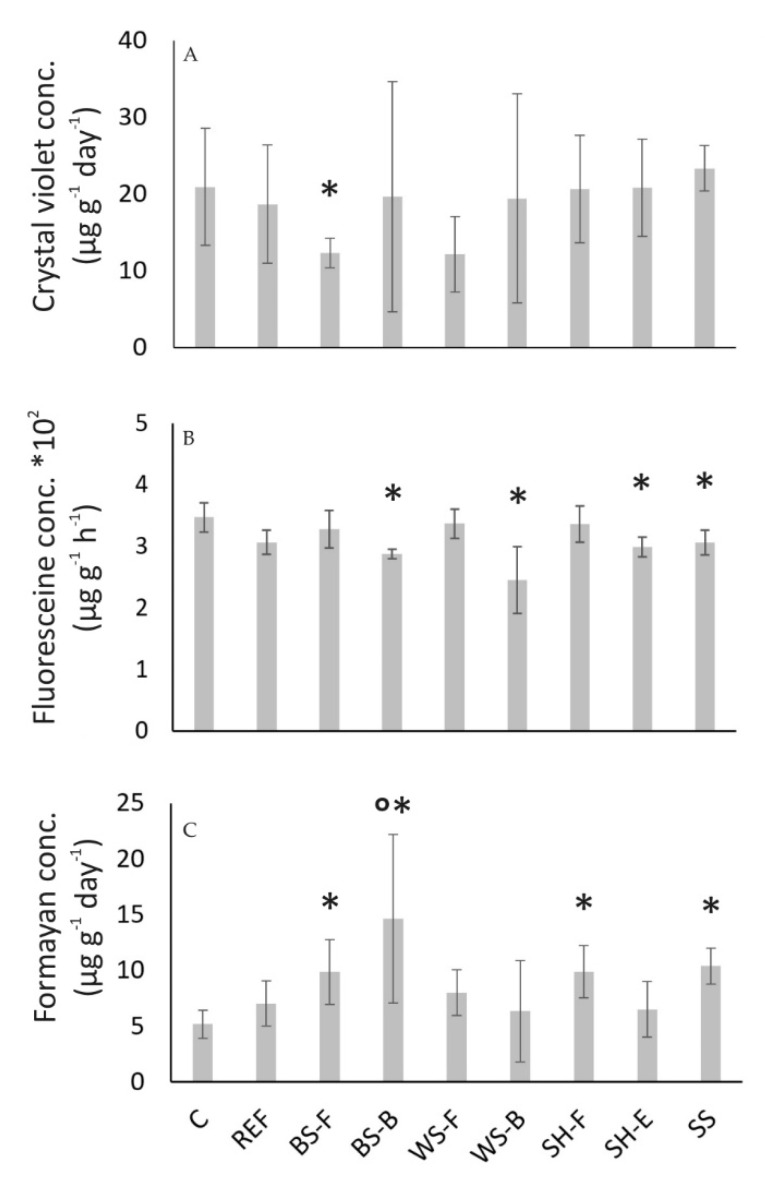
Biofilm forming ability (**A**), fluorescein diacetate activity (**B**), and dehydrogenase activity (**C**). Asterisk mark a statistically significant difference of the treatment compared to control. Circle mark a statistically significant difference of the treatment compared to the reference. All tests were significant if *p* < 0.05.

The general results of microbiological analysis suggest a low effect of leachates in tested concentrations on soil microbial activity. The BFA of the tested soil microbial community was not affected by ABA leachate as there was no significant difference between the tested groups (Kruskal–Wallis test *p* < 0.094; Figure 7A). Nevertheless, when WS-F and BS-F samples were compared directly to the control by the Mann–Whitney test, they were significantly different (BS-F, *p* < 0.008) or very close to a significant difference (WS-F, *p* < 0.056).

Such results suggest the negative effects of flying ashes of barley and wheat on biofilm formation. This is the first evidence of such effect of ABA on soil microorganisms. Barley fly ash decreased BFA by 41% compared to control. FDA test results compared between groups returned statistically significant Kruskal–Wallis test (*p* = 0.003; Figure 7B). The post-hoc comparison showed that SH-E, BS-B, WS-B and SS ashes were significantly less active compared to the control (sterile distilled water).

Moreover, the control was almost significantly different from the reference treatment (soil with lime) (K-W, *p* < 0.054), while there was no difference between the reference and the ashes. Such a result suggests the existence of a negative effect of lime toward microbial activity instead of ash [65] likely due to its high pH (>13). Hence, lime added in combination with ashes was responsible for the decrease in microbial hydrolytic activity. Nevertheless, it should be emphasized that microbial activity between control and reference decreased only by 11%. The absence of the ash effect on the FDA activity was established by other authors [66], while other authors noted stimulation of FDA hydrolysis by coal fly ash [67] or its destimulation depending on concentration [68]. The absence of the effects related to ash could be related to its small proportion in mixtures (up to 2% *w*/*w*).

DHA was also significantly different between groups (Kruskal–Wallis test *p* = 0.01; Figure 7C). There was no significant difference between control and reference treatments suggesting that stimulatory effects were indeed effects of ashes. All treatments increased dehydrogenase activity; however, only BS-B increased it significantly compared to the reference, while if compared to the control, the BS-F, SH-F and SS ashes were also significant stimulators of dehydrogenase activity. Such results are in good agreement with the literature data as many authors reported the stimulatory effect of ash on dehydrogenase activity [69,70,71] at small concentrations as a consequence of inorganic nutrient release from ash [72]. An increase in ash concentration also leads to an inhibitory effect on DHA [66,69,73,74], which was not established in this investigation.

## 4. Conclusions

The results of different previous studies have shown the potential for using different ABAs as lime substitutes in soil stabilization for road works based on the mechanical characteristics as a construction material. However, there are limited studies on the potential adverse environmental impacts within this ABA application. Based on the results of this interdisciplinary study, the following conclusions can be drawn:

- Agricultural biomass presents a good potential for use as a fuel due to measured calorific values, but higher ash content and slagging (fouling) could be expected for certain biomasses. These issues need to be analysed for each individual biomass when introducing it as a new energy source, and chemical composition and empirical indices are helpful mechanisms.
- Lime/ABA binder improved geotechnical characteristics of low-plasticity clay, and it can be potentially used as a road construction material. When evaluating the potential application of ABA within road works, the chemical composition needs to be considered since it imposes a potential application (binder substitute or filler) and chemical reactions responsible for improving strength characteristics of stabilized soil can be predicted.
- The total heavy metal content in ABA is not an appropriate measure for predicting leaching potential and potential adverse environmental influence.
- Materials embedded in road embankment and subgrade are in direct contact with the surrounding soil, and appropriate environmental risk assessment needs to be conducted. There is a lack of proper legislation addressing the limiting values of potentially harmful substances within civil construction industry. Wastewater limit values are not appropriate since imbedding material changes the environment, which highly influences the leaching potential.
- Soil microbial activity was not substantially changed. The biofilm forming ability was decreased by barley and wheat fly ashes. The hydrolytic activity was decreased by adding lime instead of ashes, while the dehydrogenase activity was stimulated by all tested ash samples.
- The amount of ABAs added to the soil for roadworks should not have adverse effects on the soil fauna in the surrounding environment.

The potential use of biomass ash depends mainly on its chemical composition, and the results and conclusions presented are limited to the ABAs used here. Future research perspectives lie in attempting to set limiting values for ABA usage in road earthworks depending on multiple criteria (the type of ABA, chemical composition of soil and ABA, lime content as a main binding agent, main purpose within pavement structure and others).

## Figures and Tables

**Table 1:** Characteristics of agricultural biomass (barley straw—BS; wheat straw—WS; sunflower seed husks—SH; and soy straw—SS).

Characteristic	Standard	Condition	BS	WS	SH	SS
Moisture content (mass%)	EN ISO 18134-1	as received	7.7	8	11.2	11.5
Ash content (mass%)	EN ISO 18122	as received	6	5.1	3.6	3.7
		dry	6.5	5.5	4	4.2
Sulfur content (mass%)	EN ISO 16994	as received	0.066	0.057	0.097	0.047
		dry	0.072	0.062	0.109	0.053
Chlorine content (mass%)	EN ISO 16994	as received	0.12	0.133	0.104	0.064
		dry	0.134	0.145	0.117	0.072
Carbon content (mass%)	EN ISO 16948	as received	42.9	42.8	46.3	42.5
		dry	46.6	46.5	52	47.7
Hydrogen content (mass%)	EN ISO 16948	as received	5.1	5.1	5.4	5.1
		dry	5.5	5.6	6.2	5.7
Nitrogen content (mass%)	EN ISO 16948	as received	0.43	0.31	0.85	0.57
		dry	0.47	0.33	0.96	0.64
Gross calorific value (MJ/kg)	EN ISO 18125	as received	17.03	17.1	18.97	16.56
		dry	18.45	18.59	21.36	18.71
Net calorific value (MJ/kg)	EN ISO 18125	as received	15.74	15.78	17.5	15.18
		dry	17.25	17.37	20.01	17.47

**Table 2:** Chemical composition of industry-generated ABAs.

Oxides (Mass%)	BS-F	BS-B	WS-F	WS-B	SH-F	SH-E	SS
P_2_O_5_	4.4	2.69	6.7	1.9	18.71	6.83	11.8
Na_2_O	0.26	0.54	0.21	0.21	<0.10	0.25	<0.10
K_2_O	44.16	7.48	32.46	17.7	24.13	43.58	8.98
CaO	10.2	7.86	15.26	6.09	28.68	18.19	41.02
MgO	4.15	2.93	7.19	2.78	21.68	11.75	21.09
Al_2_O_3_	0.68	3.01	0.86	1.24	0.52	0.52	2.5
TiO_2_	0.03	0.35	0.05	0.34	0.01	0.01	0.22
Fe_2_O_3_	0.38	1.43	0.63	0.54	0.31	0.19	1.71
SiO_2_	23.38	72.86	29.49	68.05	1.96	1.24	11.07
MnO	0.03	0.04	0.05	0.06	0.08	0.04	0.05
SO_3_	12.34	0.83	7.11	1.1	3.91	17.39	1.56

BS-F = barley straw fly ash; BS-B = barley straw bottom ash; WS-F = wheat straw fly ash; WS-B = wheat straw bottom ash; SH-F = sunflower seed husks fly ash; SH-E = sunflower seed husks economizer (heat exchanger) ash; and SS = soy straw ash.

**Table 3:** Chemical composition of laboratory-generated ABAs.

Oxide (Mass%)	BS	WS	SH	SS
P_2_O_5_	3.32	1.82	10.45	11.2
Na_2_O	0.5	0.3	1.62	<0.1
K_2_O	6.62	16.23	37.51	14.87
CaO	8.43	6.64	20.86	38.34
MgO	3.37	3.57	17.78	27.77
Al_2_O_3_	1.73	0.72	0.49	0.79
TiO_2_	0.15	0.02	1.67	0.03
Fe_2_O_3_	1.15	0.23	0.26	0.28
SiO_2_	73.39	68.97	1.58	3.3
MnO	0.04	0.07	0.05	0.03
SO_3_	1.31	1.44	7.73	3.39

BS = barley straw. WS = wheat straw. SH = sunflower seed husks. SS = soy straw ash.

**Table 4:** Tendency of biomass fuel slagging.

Empirical Indices	BS	WS	SH	SS
B/A = (Fe_2_O_3_ + CaO + MgO)/(SiO_2_ + Al_2_O_3_)	0.17-L	0.15-L	18.79-VH	16.23-VH
Sr = SiO_2_ 100/(SiO_2_ + Fe_2_O_3_ + CaO + MgO)	85.00-L	86.85-L	3.90-H	4.74-H
Al = (K_2_O + Na_2_O)/GCV	3.85-H	8.89-H	18.32-H	7.96-H
R_b/a_ = (Fe_2_O_3_ + CaO + MgO + K_2_O + Na_2_O)/(SiO_2_ + TiO_2_ + Al_2_O_3_)	0.27	0.39	20.86	19.72

slagging tendency: B/A (Acidic Compounds Ratio) < 5 Low (L); 0.5–1 Medium (M); 1–1.75 High (H); >1.75 Very high (VH). Sr (Slag Viscosity Index) > 75 Low (L); 65–75 Medium (M); and <65 High (H). Al (Alkali Index) [GJ/kg] < 0.17 Low (L); 0.17–0.34 Medium (M); and >0.34 High (H). The slagging tendency increases as R_b/a_ (Base-to-Acid Ratio) increases.

**Table 5:** Density and specific surface are (SSA) of the used materials.

Property	Soil	Lime	BS-F	BS-B	WS-F	WS-B	SH-F	SH-E	SS
Density (g/cm^3^)	2.74	2.65	2.40	2.40	2.40	2.40	2.40	2.40	2.40
SSA (m^2^/g)	0.98	1.67	3.41	3.10	3.69	3.29	3.37	4.77	10.35

**Table 6:** Heavy metal content in ABAs.

mg/kg	BS-F	BS-B	WS-F	WS-B	SH-F	SH-E	SS
As	0.04	1.26	0.11	0.25	0.04	<0.04	2.01
Cd	12.9	1.13	16.3	0.62	3.31	7.93	0.97
Cr	8.41	25.7	17.2	15.3	83.5	28.6	53.9
Pb	0.29	4.26	0.37	0.21	<0.06	0.5	10.1

**Table 7:** Heavy metals leached out of ABAs.

mg/L	BS-F	BS-B	WS-F	WS-B	SH-F	SH-E	SS
As	0.01	0.02	0.002	0.005	<0.001	<0.001	<0.001
Cd	0.002	0.001	0.002	0.009	0.01	0.002	0.0002
Cr	0.13	0.43	0.26	0.25	0.94	0.32	0.02
Pb	0.005	0.003	0.007	0.005	0.008	0.01	0.003

**Table 8:** Limit values of heavy metal emission for surface wastewater discharges.

Element	Croatian Regulation (mg/L) [58]	WHO (mg/L) [59]	US EPA (mg/L) [60]
As	0.1 *	-	0.004 to 4
Cd	0.1 *	0.003	0.0172 to 1.2
Cr-total	0.5	0.1	0.025 to 19
Pb	0.5 *	0.05	0.057 to 3.4

* pollutant whose discharge into groundwater is prohibited.

**Table 9:** Mortality rates of earthworms, pH and conductivity values of eluates in preliminary and final experiments with earthworms.

Preliminary Experiment				
ABA		Mortality (%)	Conductivity (μS/cm)	pH
Control (dH_2_O)		0	301.3	7.2
Lime		20	8352.67	13.39
SH-F		20	8448.00	13.35
SH-E		40	9160.67	13.39
BS-F		40	8837.67	13.35
BS-E		60	8882.33	13.38
BS-B		0	8056.67	13.41
WS-F		40	8885.67	13.37
WS-B		40	8141.00	13.36
SS		20	8374.00	13.33
**Final Experiment**				
**ABA**	**% of Eluate Added**	**Mortality (%)**	**Conductivity**	**pH**
Control (dH_2_O)		0	285.20	6.45
WS_F	100	0	487.13	6.68
	75	0	441.83	6.74
	50	0	419.80	6.73
	25	0	410.80	6.46
WS-B	100	0	552.80	6.75
	75	0	509.33	6.74
	50	0	421.10	6.73
	25	0	413.03	6.46
BS-E	100	0	456.40	6.83
	75	0	449.40	6.67
	50	0	415.77	6.67
	25	0	411.83	6.50
BS-F	100	0	561.40	6.81
	75	0	488.57	6.74
	50	0	449.73	6.74
	25	0	444.07	6.29
SH-E	100	0	570.70	6.75
	75	0	517.27	6.65
	50	0	498.30	6.52
	25	0	463.63	6.33
